# IL-17 and CCR9^+^α4β7^–^ Th17 Cells Promote Salivary Gland Inflammation, Dysfunction, and Cell Death in Sjögren’s Syndrome

**DOI:** 10.3389/fimmu.2021.721453

**Published:** 2021-09-01

**Authors:** Sun-Hee Hwang, Jin Seok Woo, Jeonghyeon Moon, SeungCheon Yang, Jin-Sil Park, JaeSeon Lee, JeongWon Choi, Kun Hee Lee, Seung-Ki Kwok, Sung-Hwan Park, Mi-La Cho

**Affiliations:** ^1^The Rheumatism Research Center, Catholic Research Institute of Medical Science, College of Medicine, The Catholic University of Korea, Seoul, South Korea; ^2^Department of Biomedicine & Health Sciences, College of Medicine, The Catholic University of Korea, Seoul, South Korea; ^3^Divison of Rheumatology, Department of Internal Medicine, Seoul St. Mary’s Hospital, College of Medicine, The Catholic University of Korea, Seoul, South Korea; ^4^Department of Medical Lifescience, College of Medicine, The Catholic University of Korea, Seoul, South Korea

**Keywords:** Sjögren’s syndrome, aging, interleukin 17, gut-homing, retinoic acid

## Abstract

Previous studies have evaluated the roles of T and B cells in the pathogenesis of Sjögren’s syndrome (SS); however, their relationships with age-dependent and metabolic abnormalities remain unclear. We examined the impacts of changes associated with aging or metabolic abnormalities on populations of T and B cells and SS disease severity. We detected increased populations of IL-17-producing T and B cells, which regulate inflammation, in the salivary glands of NOD/ShiLtJ mice. Inflammation-induced human submandibular gland cell death, determined based on p-MLKL and RIPK3 expression levels, was significantly increased by IL-17 treatment. Among IL-17-expressing cells in the salivary gland, peripheral blood, and spleen, the α4β7 (gut-homing integrin)-negative population was significantly increased in aged NOD/ShiLtJ mice. The α4β7-positive population markedly increased in the intestines of aged NOD/ShiLtJ mice following retinoic acid (RA) treatment. A significant increase in α4β7-negative IL-17-expressing cells in salivary glands may be involved in the onset and progression of SS. These results suggest the potential therapeutic utility of RA in SS treatment.

## Introduction

Sjögren’s syndrome (SS), first described in 1933 (1), is among the most common long-term autoimmune disorders, and is characterized by the infiltration of lymphocytes into exocrine glands, mainly the salivary and lacrimal glands, leading to the destruction of glandular tissue, followed by glandular secretion dysfunction, and both ocular dryness (keratoconjunctivitis sicca) and oral dryness (xerostomia) (2, 3).

SS has been reported to affect approximately 3–4% of adults aged 18–75 years in the general population (4). The primary symptoms of SS are dryness of the mouth, eyes, and skin and joint and muscle pain (5). Although life expectancy is unaffected by SS, it causes great inconvenience in daily life (6). SS can extend from disease confined to the exocrine glands to various extraglandular manifestations and the development of non-Hodgkin B cell lymphoma (7, 8). The pathogenesis of SS is mediated by complex mechanisms involving the infiltration of lymphocytes (mainly T and B cells) into target organs during a dysregulated adaptive immune response. T and B cell-containing ectopic lymphoid structures in the salivary and lacrimal glands include hyperactivated B cells associated with the presence of autoantibodies such as anti-SSA/Ro and anti-SSB/La autoantibodies (9). The activation of B cells by follicular helper T (T_FH_) cells is crucial for their clonal selection and affinity maturation of B cells (10).

Immune cells play crucial roles in chronic diseases related to obesity or metabolic abnormality. Altered metabolism also affects the immune system and systemic autoimmune inflammatory diseases such as rheumatoid arthritis, systemic lupus erythematosus, and gout (11, 12). The inflammatory process plays a critical role in the development of comorbidities such as hypertension, dyslipidemia, diabetes mellitus (DM), and metabolic syndrome (13). DM is the second most common metabolic disorder in SS patients, with an incidence rate 10% higher than that in the healthy population (14). DM and hypertriglyceridemia exacerbate SS *via* vascular damage followed by inflammatory processes (15). However, the pathological mechanisms of T and B cell actions in SS and metabolic disorders remain unknown.

T helper 17 (Th17) cells are crucial players in mucosal and inflammatory diseases (16). Interferon (IFN)-γ-producing Th17 cells, which are also known as Th1-like Th17 cells, have been shown to promote chronic inflammation in various autoimmune diseases and may also contribute to the pathogenesis of SS (17). Levels of IL-17, which is a characteristic cytokine of Th17 cells, are elevated in the peripheral blood of SS patients (18). Notably, vitamin A levels are significantly lower in patients with severe SS than in those with mild SS (19). Retinoic acid (RA) is a metabolite of vitamin A that mediates human growth and development (20). It plays a major role in the pathophysiology of inflammation and has an immunosuppressive effect in autoimmune diseases (21, 22). RA is an important regulatory factor for the induction of immune tolerance in the intestine (23) and has been reported to regulate reciprocal differentiation of regulatory T (Treg) cells and Th17 cells (24). It has been shown that RA has immunosuppressive effect on Th1/Th17 cells in multiple sclerosis (MS) (25). In addition, RA can repress the expression of inflammatory chemokines and cytokines, inhibiting inflammatory responses triggered by obesity (26). It shows a potential modulatory role of RA in metabolic diseases. Vitamin A is metabolized into RA in the intestine, and RA regulates important signaling pathways in the intestinal environment (27, 28). RA-induced gut-homing molecules such as CCR9 and α4β7 show tissue tropism of T cells for migration into the small intestine (29–31). Administration of α4β7 blocking-antibodies increased the peripheral availability of Th17 cells, resulting in increased experimental autoimmune encephalomyelitis (EAE) secerity (32). However, the role of RA in the immune response in SS has not been elucidated. Thus, RA is an important regulatory factor inducing immune tolerance in the intestines of SS patients. In the present study, we hypothesized that RA promotes anti-inflammatory factors and suppresses proinflammatory factors in SS patients with spontaneous type 1 diabetes (T1D).

In a recent study, we found that SS symptoms were more severe when accompanied by metabolic abnormalities, such as DM; invasion of Th17 and T_FH_17 cells into spleen and salivary gland tissues was dramatically increased in SS mice with DM (33). In the present study, we investigated whether Th17 cells express CCR9 and α4β7, which are a chemokine receptor and an integrin of intestinal origin, respectively. We also explored whether homing from the periphery to the intestine is helpful for improving SS symptoms in aged NOD/ShiLtJ mice following RA treatment. Finally, we compared IL-17 expression levels between young and old mice.

## Materials and Methods

### Animals

We purchased 7-week-old female NOD/ShiLtJ mice from Jackson Laboratories (Bar Harbor, ME, USA). The mice were housed under specific-pathogen-free conditions at the Catholic Research Institute of Medical Science, Catholic University of Korea, and were fed a gamma ray-sterilized diet (TD 2018S; Harlan Laboratories, Tampa, FL, USA) and autoclaved water. All animal procedures were performed in accordance with the Laboratory Animals Welfare Act, the Guide for the Care and Use of Laboratory Animals, and the Guidelines and Policies for Rodent Experiments provided by the Institutional Animal Care and Use Committee of the School of Medicine, The Catholic University of Korea (approval no.: CUMS-2020–0271–01).

### Patients

Patients were diagnosed with pSS, according to the American–European Consensus Group criteria for pSS or the 2012 American College of Rheumatology criteria. Informed consent was obtained from all patients according to the principles of the Declaration of Helsinki. Serum samples were obtained from age/sex-matched healthy volunteers, who served as controls. This study was approved by the Institutional Review Board of Seoul St. Mary’s Hospital (KC13ONMI0646).

### Measurement of Blood Glucose and Salivary Secretion in NOD/ShiLtJ Mice

Mice were anesthetized by inhalation of isoflurane (2%), and blood glucose levels were determined using an Accu-Check Compact glucometer (Roche, Indianapolis, IN, USA). Whole saliva was collected for 7 min from the oral cavity, starting at 90 s after intraperitoneal injection of pilocarpine (100 μg/mouse; Sigma-Aldrich, St. Louis, MO, USA). Saliva flow rates were expressed as μL saliva secreted per g body weight per min (μL/g/min).

### Histopathological Assessment of Inflammation

Tissues were fixed in 10% formalin and embedded in paraffin. Sections were stained with hematoxylin and eosin (H & E) and Masson’s trichrome. Salivary gland and small intestine inflammation were scored as previously described (34, 35). Scoring criteria for salivary gland: score 0, no infiltrates; score 1–1.5, 1–2 foci per section; 2–2.5, 3–5 foci per section; score 3, 6–10 foci per section; score 4, more than 10 foci per section. Immunohistochemistry was analyzed using Vectastain ABC kits (Vector Laboratories, Burlingame, CA, USA). Tissue sections were first incubated with primary antibodies to IL-6 and IL-17 (Abcam, Cambridge, UK) overnight at 4°C, each primary antibody was detected using a biotinylated secondary antibody, followed by incubation with streptavidin–peroxidase complex for 1h. DAB chromogen (Dako, Carpinteria, CA, USA) was added as the substrate. Double immunohistochemistry was performed using Polink DS-MR-Ms A Double IHC staining kits (GBI Labs, Mukilteo, WA, USA). The stained cells were visualized by microscopy (Olympus, Center Valley, PA, USA).

### Intracellular Staining and Flow Cytometry

Cells were isolated from spleens, salivary glands, and peripheral blood of NOD/ShiLtJ mice, and stimulated with 25 ng/mL phorbol myristate acetate and 250 ng/mL ionomycin (Sigma-Aldrich) in the presence of GolgiStop (BD Biosciences, San Jose, CA, USA) for 4 h. The cells were stained with surface PerCP or PB450-conjugated anti-CD4, APC-conjugated anti-CD25, PerCP-conjugated anti-C-X-C chemokine receptor type 5 (CXCR5), PE-Cy7-conjugated anti-Inducible T-cell COStimulator (ICOS), and PE-Cy7-conjugated anti-CD19 (eBioscience, San Diego, CA, USA) antibodies. Gut-homing molecules were stained with surface phycoerythrin (PE)-conjugated anti-α4β7 and allophycocyanin (APC)-conjugated anti-CCR9 antibodies (eBioscience). Surface-labeled cells were permeabilized using Cytofix/Cytoperm solution (BD Pharmingen, Franklin Lakes, NJ, USA), and then intracellular staining for IL-17 was performed using PE or Fluorescein isothiocyanate (FITC)-conjugated anti-IL-17 and PE-conjugated anti-Foxp3 (eBioscience). All samples were analyzed using the FACS Calibur (BD Pharmingen), fluorescence-activated cell sorting (FACS) instrument, and data were analyzed using the FlowJo software (Tree Star, Ashland, OR, USA).

### Microarray Analysis

Microarray analysis was performed using the Affymetrix Mouse Gene 2.0 ST Array by (Macrogen Co., Seoul, Korea) according to the manufacturer’s instructions. Differentially expressed genes from salivary gland cells were compared between 21-week-old NOD/ShiLtJ mice and NOD/ShiLtJ mice with T1D (Accession Number: GSE179654).

### Western Blotting

A human submandibular gland (HSG) cell line was cultured with recombinant human TNF-α (2 ng/mL) and in the presence or absence of recombinant human IL-17 (20 or 40 ng/mL) for 48 h, and cell lysates were prepared. The protein concentration was determined using the Bradford method (Bio-Rad, Hercules, CA, USA), and samples were separated by sodium dodecyl sulfate–polyacrylamide gel electrophoresis (SDS–PAGE) and transferred to nitrocellulose membranes (Amersham Pharmacia, Uppsala, Sweden). Primary antibodies to p-MLKL (Abcam), RIPK3, and β-actin (Santa Cruz Biotechnology, Dallas, TX, USA) were diluted with 0.1% skim milk in Tris-buffered saline and incubated for 20 min at room temperature. The membranes were washed and incubated with horseradish peroxidase-conjugated secondary antibody for 15 min at room temperature.

### Isolation and Culture of SGSCs

Sphere-forming murine SGSCs were isolated from mouse submandibular glands using a previously described method (36). The thin fascia covering the submandibular glands were carefully removed to expose the submandibular tissues. The glands were washed three times in phosphate-buffered saline (PBS, Gibco, Grand Island, NY, USA) with 3% penicillin/streptomycin and then chopped into small tissue fragments, which were maintained at 37°C in Dulbecco’s modified Eagle’s medium (DMEM) containing 1 mg/mL collagenase I (Gibco) under an atmosphere of 5% CO_2_ for 30 min. The fragments were then centrifuged, and the pellets were resuspended in DMEM. The cells were filtered through a 40 μm cell strainer (BD Pharmingen) to produce a single-cell suspension. After centrifugation, the cells were cultured in DMEM/F12 culture medium (1:1 mixture, v/v; Gibco) supplemented with 20 ng/mL epidermal growth factor (PeproTech, Rocky Hill, NJ, USA), 20 ng/mL fibroblast growth factor-2 (PeproTech), 1% N-2 Supplement (Gibco), 1% insulin–transferrin–selenium (Gibco), 1 μm dexamethasone (Sigma-Aldrich), and 1% penicillin/streptomycin (Sigma-Aldrich). SGSCs were cultured in Matrigel (Corning Inc., Corning, NY, USA) or suspension culture for 1 week. SGSCs were treated with 10 or 20 ng/mL IL-17.

### α-Amylase Assay

SGSC α-amylase activity was determined using an α-amylase assay kit (Abcam) according to the manufacturer’s instructions.

### Real-Time Polymerase Chain Reaction (qPCR)

mRNA was extracted using TRI Reagent (Molecular Research Center, Cincinnati, OH, USA) according to the manufacturer’s instructions. PCR amplification was performed using the Applied Biosystems StepOne Plus real-time PCR system (Applied Biosystems, Foster City, CA, USA). All reactions were performed using SensiFAST SYBR Hi-ROX (Bioline USA Inc., Taunton, MA, USA) according to the manufacturer’s instructions. The following primers were used to amplify the human genes (37, 38): Aquaporin 5 (AQP5), 5′-GCC CTC TTA ATA GGC AAC CAG-3′ (sense) and 3′-GCA TTG ACG GCC AGG TTA C-5′ (antisense); amylase1 (Amy1), 5′-AAC CCA AAT AAC AGG GAC TTT CC-3′ (sense) and 3′-GGT AGT TCT CGA TAC CTC CAC TT-5′ (antisense); keratin18 (Krt18), 5′-ACT CCG CAA GGT GGT AGA TGA-3′ (sense) and 3′-TCC ACT TCC ACA GTC AAT CCA-5′ (antisense); and Nanog, 5′-CAC AGT TTG CCT AGT TCT GAG G-3′ (sense) and 3′-GCA AGA ATA GTT CTC GGG ATG AA-5′ (antisense); β-actin, 5′-GAA ATC GTG CGT GAC ATC AAA G-3′ (sense) and 3′-TGT AGT TTC ATG GAT GCC ACA G-5′ (antisense). All expression values were normalized to β-actin expression in the same RNA sample and calculated using the 2^–ΔΔCt^ method.

### Cell Isolation and Culture

Splenic CD4^+^ T cells were isolated from 6-week-old NOD/ShiLtJ mice. For Th17 cell differentiation, the cells were stimulated with anti-CD3 (0.5 µg/mL), anti-CD28 (1 µg/mL), anti-IFN-γ (10 µg/mL) and anti-IL-4 (10 µg/mL) antibodies, IL-6 (20 ng/mL), and transforming growth factor-β (TGF-β) (2 ng/mL) for 3 days. Recombinant mouse IL-6 and antibodies to IFN-γ and IL-4 were purchased from R&D Systems (Minneapolis, MN, USA), and TGF-β was purchased from PeproTech. Cells were pretreated with RA (Sigma-Aldrich, St. Louis, MO, USA) at concentrations of 0.2–1 μM for 2 h and then stimulated under the required conditions.

### ELISA

Serum samples were stored at –20°C until use. Total IgA levels in sera were measured using IgA ELISA quantification kits (Bethyl Lab Co., Montgomery, TX, USA). The IL-17 levels in culture supernatants were determined using sandwich ELISA (DuoSet; R&D Systems, Lille, France). Horseradish peroxidase-conjugated streptavidin (HRP-Streptavidin) was used for color development. Serum levels of RA were determined using an RA ELISA kit (MyBioSource, San Diego, CA, USA). Horseradish peroxidase-conjugated RA (HRP-RA) was used for color development. Absorbance at 450 nm (A_450_) was measured on an ELISA microplate reader (Molecular Devices, Sunnyvale, CA, USA).

### Confocal Microscopic Analysis

Small intestine tissues were stained with anti-CD4 (Santa Cruz Biotechnology, Dallas, TX, USA), anti-IL-17 (Abcam), and anti-α4β7 (Biolegend, San Diego, CA, USA) primary antibodies at 4°C overnight, followed by secondary antibodies conjugated with FITC (Santa Cruz Biotechnology, APC (Thermo Fisher Scientific, Rockford, IL, USA) and PE (Thermo Fisher Scientific) incubated at room temperature for 2 h. Nuclei were stained with 4,’6-diamidino-2-phenylindole (DAPI; Invitrogen, Carlsbad, CA). Confocal images were analyzed using an LSM 700 confocal microscope (Zeiss, Oberkochen, Germany) at 200× magnification.

### RA Treatment

We injected 12-week-old NOD/ShiLtJ mice intraperitoneally with 1 mg/kg RA dissolved in corn oil three times per week for 6 weeks. Control mice received intraperitoneal injection of corn oil according to the same schedule.

### Statistical Analyses

Data are presented as means ± standard errors of the mean (SEM). All statistical analyses were performed using the GraphPad Prism ver. 5 software for Windows (GraphPad Software, San Diego, CA, USA). Normally distributed continuous data were analyzed using the parametric Student’s *t-*test. Differences in means among groups were subjected to one-way analysis of variance (ANOVA). In all analyses, *P* < 0.05 was taken to indicate statistical significance.

## Results

### Blood Glucose and IL-17 Expression Levels Are Higher in NOD/ShiLtJ Mice With Aging and T1D Development

**Figure 1:**
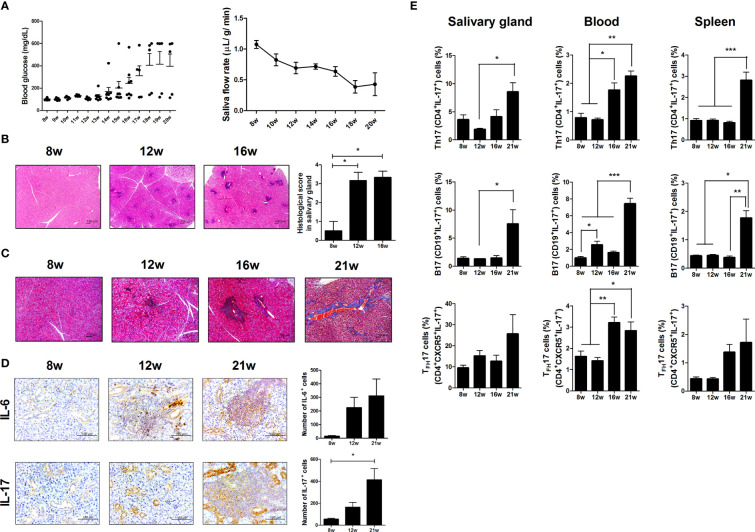
Investigation of Sjögren’s syndrome (SS) and T1D symptoms in NOD/ShiLtJ mice. **(A)** Blood glucose levels (left) and salivary flow rates (right) were measured in NOD/ShiLtJ mice at the indicated ages (N=20 at 8 wk, 15 at 9 to12 wk, 10 at 13 to16 wk, and 5 at 17 to 20 wk). **(B)** Representative hematoxylin and eosin (H&E)-stained images of salivary glands from NOD/ShiLtJ mice at the indicated ages. Scale bar = 100 µm. Bar graph shows average histological scores (N=5). **(C)** Representative Masson’s trichrome-stained images of salivary glands from NOD/ShiLtJ mice at the indicated ages (N=5). Scale bar = 100 µm. **(D)** Representative IL-6- (top) and IL-17- (bottom) stained images of salivary glands of NOD/ShiLtJ mice at the indicated ages. Bar graphs show average numbers of IL-6- (top) and IL-17- (bottom) positive cells (N=5). **(E)** Bar graphs show average frequencies of CD4^+^IL-17^+^ (Th17), CD19^+^IL-17^+^ (B17), and CD4^+^CXCR5^+^IL-17^+^ (T_FH_17) cells among cells isolated from salivary glands (left), peripheral blood (center), and spleens (right) of NOD/ShiLtJ mice at the indicated ages (N=5). Cells were stimulated with phorbol 12-myristate 13-acetate (PMA) and ionomycin for 4 h and GolgiStop for the final 2 h, and then stained with the indicated antibodies for flow cytometry analysis. Values are means ± SEM from three independent experiments. *p < 0.05, **p < 0.01, ***p < 0.001.

To investigate the effects of aging and metabolic alterations on SS progression, we compared metabolic alteration and SS progression in differnet aged NOD/ShiLtJ mice. Blood glucose levels were higher in NOD/ShiLtJ mice with T1D (**Figure 1A** and **Supplementary Figure 1A**). NOD/ShiLtJ mice with T1D also showed body weight loss (**Supplementary Figure 1A**). Salivary secretion decreased in an age-dependent manner in mice with T1D (**Figure 1A** and **Supplementary Figure 1B**). Lymphocytic infiltration into the salivary glands gradually increased between 8 and 16 weeks, and fibrosis occurred at 21 weeks (**Figure 1B, C** **)**. Focal inflammation and fibrosis of the salivary gland were exacerbated in NOD/ShiLtJ mice with T1D (**Supplementary Figures 1C, D** **)**. The infiltration of proinflammatory cytokines, such as IL-6 and IL-17 into the salivary glands increased with age (**Figure 1D**) or metabolic abnormalities (**Supplementary Figure 1E**). Flow cytometric analysis showed that Th17 (CD4^+^IL-17^+^), T_FH_17 (CD4^+^CXCR5^+^ICOS^+^IL-17^+^), and B17 (CD19^+^IL-17^+^) cells increased with age or T1D in salivary gland cells, peripheral blood mononuclear cells, and splenocytes (**Figure 1E** and **Supplementary Figure 1F**). These data suggest that increased proliferation of IL-17-producing cells causes inflammation in salivary gland tissue and exacerbates SS symptoms.

### IL-17 Causes Inflammation-Induced Cell Death and Inhibited Self-Renewal in Salivary Gland Stem Cells (SGSCs)

**Figure 2:**
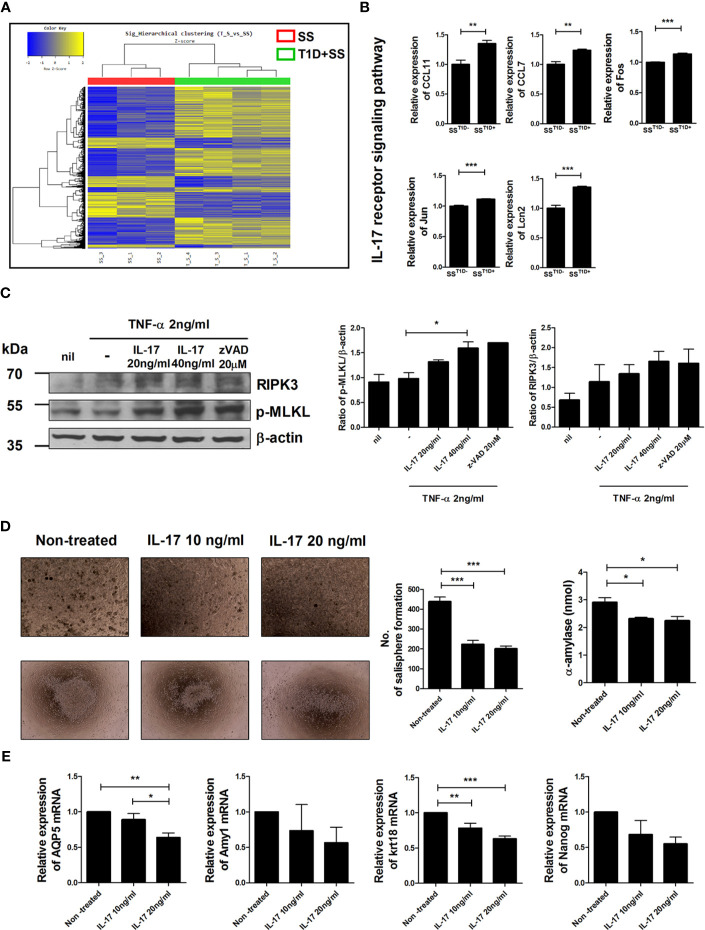
Effects of IL-17 in salivary gland cells. **(A)** Hierarchical clustering shows gene expression in isolated salivary gland cells from NOD/ShiLtJ mice with or without T1D. Yellow and blue regions in cells indicate high and low relative expression levels, respectively. **(B)** Bar graphs show expression levels of indicated genes involved in the IL-17 receptor signaling pathway according to microarray analysis. **(C)** Human salivary gland cells were cultured with TNF-α in the absence or presence of recombinant human IL-17 or z-VAD for 48 h, and the expression levels of p-MLKL (left), RIPK3 (right), and β-actin were examined by Western blotting. Bar graphs show average expression levels of p-MLKL (left) and RIPK3 (right). **(D)** Micrographs show mouse salisphere sizes in cultured salivary gland stem cells (SGSCs) with IL-17. Original magnifications were 200× (top) and 40× (bottom). Bar graphs show average numbers of salispheres (left) and average secretion of α-amylase (right) under the indicated conditions. **(E)** Bar graphs show average transcription levels of *AQP5*, *Amy1*, *Krt18*, and *Nanog* under the indicated conditions. Values are means ± SEM from three independent experiments. *p < 0.05, **p < 0.01, ***p < 0.001.

Profiles of gene expression in salivary gland cells were compared between NOD/ShiLtJ mice with or without T1D (**Figure 2A**). Among 41,345 analyzed genes, 1,051 were upregulated and 695 were downregulated in NOD/ShiLtJ mice with T1D compared with those without T1D. The expression of IL-17 receptor signaling pathway-related genes such as CCL11, CCL7, Fos, Jun, and Lcn2, was elevated in NOD/ShiLtJ mice with T1D (**Figure 2B**). These data suggest that activation of the IL-17 signaling pathway accelerates SS progression. To determine the effects of IL-17 on salivary glands, we investigated the expression levels of the inflammatory cell death-related markers p-MLKL and RIPK3 in IL-17-treated human submandibular gland (HSG) cells. The expression levels of p-MLKL and RIPK3 increased similarly to those under necroptotic conditions (TNF-α + z-VAD) after IL-17 treatment (**Figure 2C**). We also investigated the effects of IL-17 on the self-renewal and differentiation potential of SGSCs. Salisphere morphology numbers were used as an indicator of SGSC self-renewal potential. Following IL-17 treatment, the salisphere population and α-amylase activity of SGSCs were reduced (**Figure 2D**) and the transcript levels of *AQP5*, *Amy1*, *Krt18*, and *Nanog* were decreased in SGSCs (**Figure 2E**). These data suggest that IL-17 caused inflammation-induced cell death in salivary gland cells and downregulated the stem cell properties of SGSGs.

### Th17 Cells Expressing Gut-Homing Molecules Decrease With Age in NOD/ShiLtJ Mice

**Figure 3:**
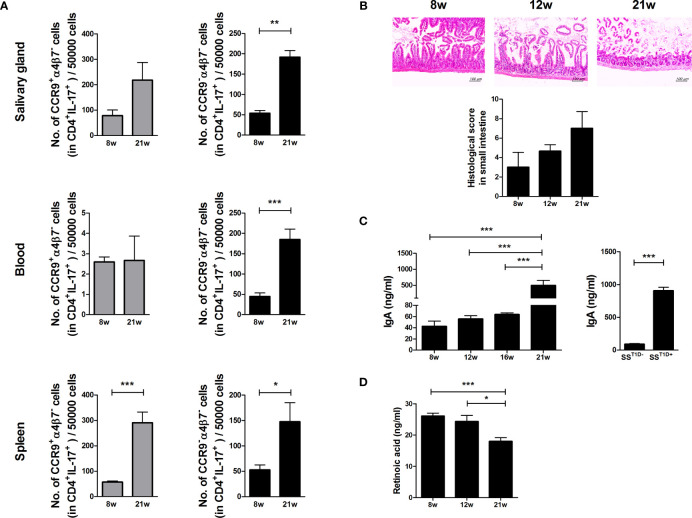
Regulation of small intestinal inflammation and gut-homing molecules expression in Th17 cells due to retinoic acid (RA) deficiency in aged mice. **(A)** Bar graphs show average numbers of CCP9^+^α4β7^–^ (left) and CCP9^–^α4β7^–^ (right) cells among cells isolated from salivary glands (top), peripheral blood (middle), and spleens (bottom) of 8- and 21-week-old NOD/ShiLtJ mice. Cells were stimulated with PMA and ionomycin for 4 h and GolgiStop for the final 2 h, and then stained with the indicated antibodies for flow cytometry analysis (N=5). **(B)** Representative H&E-stained images of small intestines from NOD/ShiLtJ mice at the indicated ages. Bar graph shows average histological scores (N=5). Scale bar= 100 µm. **(C)** Bar graphs show average IgA levels in sera from NOD/ShiLtJ mice at the indicated ages (left) and SS mice with or without T1D (right) (N=5). **(D)** Bar graph shows average retinoic acid levels in sera from NOD/ShiLtJ mice at the indicated ages (N=5). Values are means ± SEM from three independent experiments. *p < 0.05, **p < 0.01, ***p < 0.001.

To investigate whether Th17 cells return to the gut, the expression levels of gut-homing molecules such as CCR9 and α4β7 were examined in Th17 cells of the salivary glands, peripheral blood, and splenocytes of NOD/ShiLtJ mice. The numbers of α4β7^-^ Th17 cells increased in the salivary glands, peripheral blood, and splenocytes of aged NOD/ShiLtJ mice (**Figure 3A**) and intestinal damage severity increased in an age-dependent manner (**Figure 3B**). IgA levels were significantly higher in 21-week-old mice and mice with T1D (**Figure 3C**). These data suggest that intestinal damage is caused by an imbalance between intestinal tolerance and increased serum levels of IgA. RA plays a crucial role in the pathophysiology of inflammation. To investigate the role of RA in SS, we measured RA levels in NOD/ShiLtJ mice and found that RA levels gradually decreased in an age-dependent manner (**Figure 3D**).

### RA Regulates the Expression of IL-17 and Gut-Homing Molecules *In Vitro*

**Figure 4:**
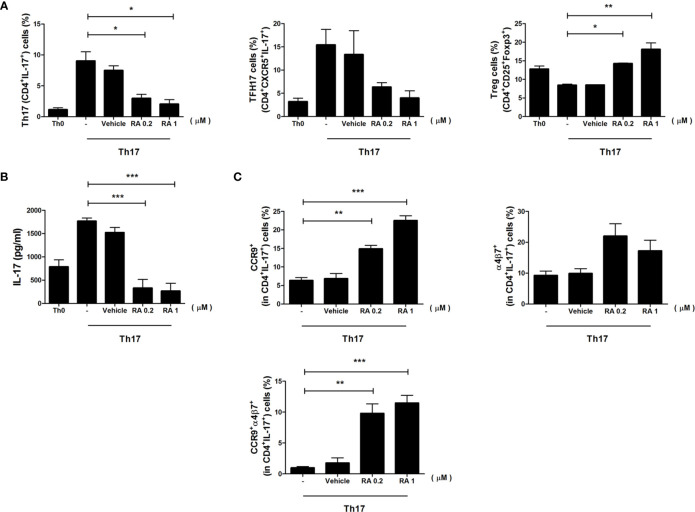
The role of RA in the regulation of CD4+ T cells and gut-homing molecules. Splenic CD4^+^ T cells were isolated from NOD/ShitJ mice and then cultured under Th17 differentiation conditions with or without RA for 3 days. **(A)** Bar graphs show average frequencies of CD4^+^IL-17^+^ (Th17), CD4^+^CXCR5^+^ IL-17^+^ (T_FH_17), and CD4^+^CD25^+^Foxp3^+^ (Treg) cells under the indicated conditions. **(B)** Bar graph shows average levels of secretory IL-17, as determined in the culture supernatant by enzyme-linked immunosorbent assay (ELISA) under the indicated conditions. **(C)** Bar graphs show average frequencies of CCR9^+^ (top and left), α4β7^+^ (top and right), and CCR9^+^α4β7^+^ (bottom) in CD4^+^IL-17^+^ cells under the indicated conditions. Data are means ± SEM from three independent experiments. *p < 0.05, **p < 0.01, ***p < 0.001.

To determine the role of RA in the induction of gut-homing molecules expression in Th17 cells, we sorted CD4^+^ T cells from spleen and cultured cells under Th17 differentiation with RA for 3 days. The number of Th17 cells decreased significantly, whereas the number of Treg cells increased in the RA treatment group (**Figure 4A**). IL-17 secretion was examined in the culture supernatants using an enzyme-linked immunosorbent assay (ELISA) and found to be lower in the RA treatment groups (**Figure 4B**). Interestingly, RA increased the expression levels of gut-homing molecules CCR9 and α4β7 in Th17 cells, and populations of CCR9 and α4β7 double-positive Th17 cells increased markedly in the RA treatment groups (**Figure 4C**). These data demonstrate that RA regulated the numbers of Treg cells and Th17 cells expressing gut-homing molecules.

### RA Administration Regulates Blood Glucose, Salivary Secretion, and Inflammation in the Salivary Glands and Small Intestines of NOD/ShiLtJ Mice

**Figure 5:**
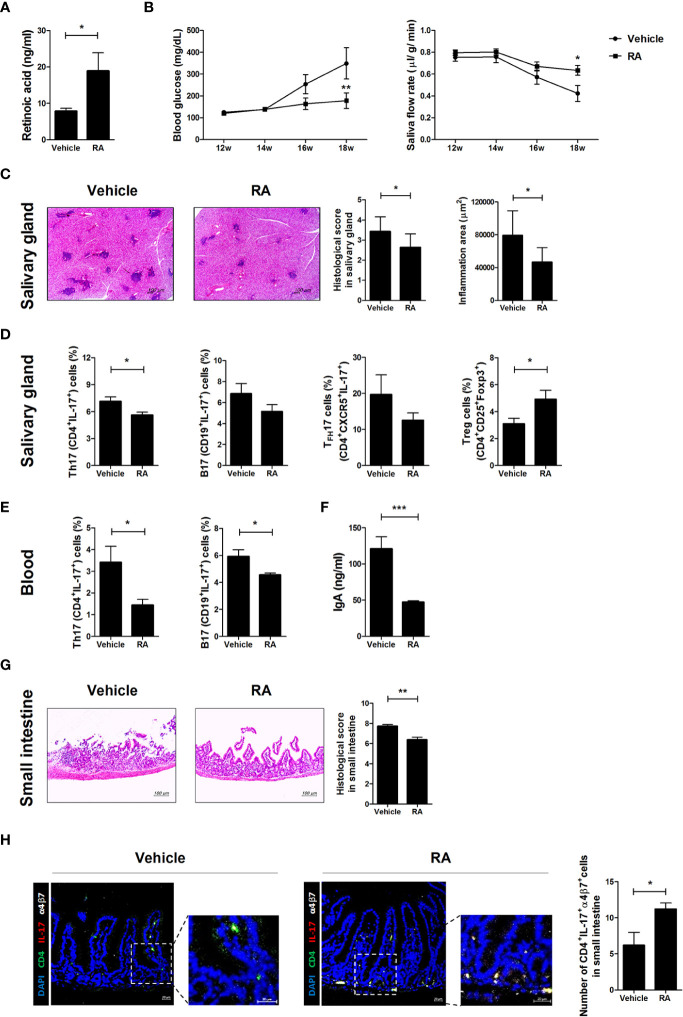
Treatment with RA ameliorated SS symptoms in NOD/ShiLtJ mice. We injected 12-week-old NOD/ShiLtJ mice intraperitoneally with 1 mg/kg RA three times per week for 6 weeks. **(A)** Bar graph shows average RA levels in sera from vehicle- (N=5) and RA-treated mice (N=5). **(B)** Blood glucose levels (left) and salivary flow rates (right) were measured in vehicle- (N=10) and RA treated-NOD/ShiLtJ mice (N=10) at the indicated ages. **(C)** Representative H&E-stained images of salivary glands from vehicle- (left) (N=10) and RA treated- (right) NOD/ShiLtJ mice (N=10). Bar graph shows average inflammation area of salivary glands. Scale bar= 100 µm. **(D)** Bar graphs show average frequencies of Th17 (CD4^+^IL-17^+^), B17 (CD19^+^IL-17^+^), T_FH_17 (CD4^+^CXCR5^+^IL-17^+^), and Treg (CD4^+^CD25^+^Foxp3^+^) cells among cells isolated from salivary glands of vehicle- (N=10) and RA-treated NOD/ShiLtJ mice (N=10). Cells were stimulated with PMA and ionomycin for 4 h and GolgiStop for the final 2 h, and then stained with the indicated antibodies for flow cytometry analysis. **(E)** Bar graphs show average frequencies of Th17 (CD4^+^IL-17^+^) and B17 (CD19^+^IL-17^+^) cells in peripheral blood mononuclear cells of vehicle- (N=10) and RA-treated NOD/ShiLtJ mice (N=10). Cells were stimulated with PMA and ionomycin for 4 h and GolgiStop for the final 2 h, and then stained with the indicated antibodies for flow cytometry analysis. **(F)** Bar graph shows average IgA levels in sera from vehicle- and RA-treated mice. **(G)** Representative H & E-stained images of small intestines from vehicle- (left) (N=10) and RA treated- (right) NOD/ShiLtJ mice (N=10). Bar graph shows average histological scores for small intestine tissues. Scale bar= 100 µm. **(H)** Representative immunofluorescence images for CD4 (green), IL-17 (red), α4β7 (white) and DAPI counterstaining (blue) in small intestine tissues of vehicle- (N=5) and RA-treated mice (N=5). Original magnification was 200×. Values are means ± SEM from three independent experiments. *p < 0.05, **p < 0.01, ***p < 0.001.

To investigate whether RA ameliorates SS symptoms in NOD/ShiLtJ mice, RA was injected intraperitoneally three times per week at a dose of 1 mg/kg into NOD/ShiLtJ mice. RA levels were significantly higher in the sera of RA-treated mice (**Figure 5A**). Blood glucose levels were lower and the salivary flow rate was higher in the RA-treated group than in the vehicle-treated group (**Figure 5B**). RA also attenuated the infiltration of lymphocytes into the salivary glands compared with the vehicle-treated group (**Figure 5C**). The numbers of Th17 (CD4^+^IL-17^+^), T_FH_17 (CD4^+^CXCR5^+^IL-17^+^), and B17 (CD19^+^IL-17^+^) cells were lower in the salivary glands of RA-treated mice, whereas those of Treg (CD4^+^CD25^+^Foxp3^+^) cells were higher in the salivary glands of RA-treated mice (**Figure 5D**). The numbers of Th17 (CD4^+^IL-17^+^) and B17 (CD19^+^IL-17^+^) cells were lower in the peripheral blood mononuclear cells of RA-treated mice (**Figure 5E**). IgA levels were significantly lower in the sera of RA-treated mice (**Figure 5F**) and the numbers of α4β7^-^ Th17 cells were lower in the salivary glands and spleens of RA-treated mice (**Supplementary Figure 2A**). Furthermore, small intestine damage was ameliorated by RA treatment (**Figure 5G**) and α4β7^+^ Th17 cell proliferation increased in the RA-treated group (**Figure 5H**). These data suggest that RA ameliorated SS symptoms and high blood glucose by reducing the infiltration of IL-17-producing cells into the salivary gland and increasing the numbers of gut-homing Th17 cells in the small intestine. Levels of serum IgA, a factor involved in the intestinal barrier were reduced in RA-treated mice, alleviating barrier damage.

### IL-17 Levels Are Higher and RA Levels Are Lower in SS Patients

**Figure 6:**
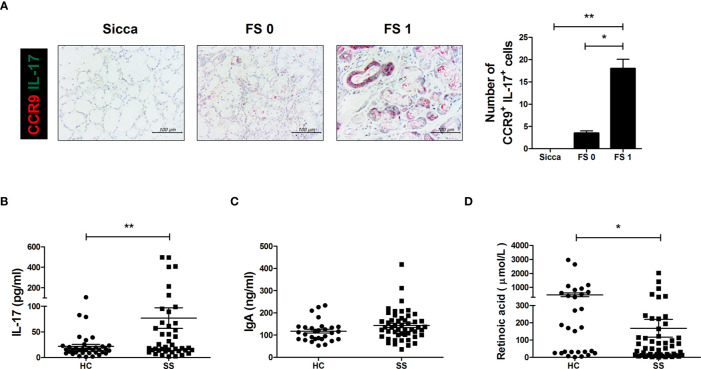
IL-17 cells expressing gut-homing receptors in salivary glands and RA, IgA, and IL-17 levels in SS patient sera. **(A)** Representative CDR9- and IL-17-stained salivary glands from the indicated patient groups. Bar graph shows average numbers of CCR9^+^IL-17^+^ cells in salivary gland tissues. **(B–D)** Bar graphs show average levels of IL-17 **(B)**, IgA **(C)**, and RA **(D)** in sera from healthy control (HC) and SS patients. Values are means ± SEM. *p < 0.05, **p < 0.01.

Based on these findings, we examined CCR9 and IL-17 expression in human salivary gland biopsy specimens acquired from non-SS patients and patients with a histological score of either 0 or 1. Higher scores were associated with greater numbers of CCR9 and IL-17 double-positive cells in salivary gland tissues (**Figure 6A**). IL-17 production was significantly higher in sera from SS patients than in those of healthy controls (**Figure 6B**) and IgA levels were generally higher in SS patient sera (**Figure 6C**). As expected, RA concentration was lower in sera from SS patients than in healthy controls (**Figure 6D**). These findings demonstrate the therapeutic potential of RA in SS patients.

## Discussion

Lymphoid follicles consist of Peyer’s patches and isolated lymphoid follicles beneath the intestinal epithelium. Within the follicles, a variety of immune cells including B and T cells, dendritic cells, and neutrophils regulate immune responses by presenting antigens, secreting cytokines, and producing antigen-binding antibodies (39). Another component of the immunological barrier is secretory IgA, which is present primarily on intestinal mucosal surfaces and is an important factor for interactions with commensal bacteria to protect against pathogens (40). Recent studies have shown that intestinal barrier dysfunction may be an important causative factor in autoimmune diseases (41, 42). Some studies have demonstrated a role of IL-17 in the pathogenesis of SS (43, 44). IL-17 has been reported to promote SS pathogenesis in an age-dependent manner, but this process has not been examined in detail.

Aging is characterized by a loss of cellular function, which is associated with the loss of adaptive response to stress and increasing probability of death (45). Aging leads to metabolic abnormalities, which can include DM, and biological changes in the immune system (46). Most SS patients are around 50 years of age at the time of diagnosis (47). In this study, we investigated the effects of metabolic abnormalities related to age on immune cells in NOD/ShiLtJ mice with SS and T1D. We found that blood glucose levels increased and salivary flow rates decreased with aging. Infiltration of lymphocytes into the salivary glands and salivary gland fibrosis were more prevalent among aged mice. Furthermore, greater numbers of IL6- and IL-17-producing cells infiltrated the salivary glands, and greater numbers of Th17, T_FH_17, and B17 cells were observed in the salivary glands, peripheral blood, and spleen with aging. Among aged NOD/ShiLtJ mice, we observed lower body weight and salivary flow rates and higher blood glucose levels, and infiltration of inflammatory cells into the salivary glands in mice with spontaneous T1D than in those without T1D. NOD/ShiLtJ mice with spontaneous T1D showed higher infiltration of IL-6- and IL-17-producing cells into the salivary glands than in mice without T1D. Furthermore, more Th17 and B17 cells were observed in the salivary glands, peripheral blood, and spleen. These data suggest that metabolic abnormalities associated with aging may contribute to the development of SS by increasing infiltration of IL-17-producing immune cells into the salivary glands.

The expression of IL-17 receptor signaling pathway-related genes was significantly higher in the salivary gland cells of NOD/ShiLtJ mice with spontaneous T1D. There have been several reports that TNF-α and IFN-γ induce apoptosis in HSG cells (25, 48). However, there were no reports about role of IL-17 in cell death of HSG. In this study, we investigated the role of IL-17 in cell death of HSG. The necroptotic markers (49), p-MLKL and RIPK3, were highly expressed in IL-17-treated salivary gland cells. Salisphere formation, saliva secretion, and transcript levels of a salivary functional gene (*Amy1*), acinar cell marker (*AQP5*), ductal cell marker (*Krt18*), and stem cell marker (*Nanog*) (37) were lower in IL-17-treated SGSCs. These data suggest that increased IL-17 expression induced by metabolic abnormalities may cause cell death and inhibit tissue regeneration in the salivary glands of SS patients.

RA is a critical factor for maintaining intestinal homeostasis by directly modulating effector cytokines (22). Integrin α4 and integrin β7 form gut-homing integrin α4β7 in T cells. Recent studies have shown that the regulation of homing molecules during T cell activation affects T cell function in a variety of positive and negative signals (50, 51). Suppression or upregulation of RA causes differential expression of gut-homing molecules (CCR9 and α4β7) in Th17 cells (52). Our results, based on *in vitro* and *in vivo* experiments and data from SS patients, suggest that decreased RA levels exacerbate SS by retaining IL-17-producing immune cells in the peripheral blood, salivary glands, and spleen instead of their migration to the gut.

In conclusion, the inflammation of salivary gland tissue in NOD/ShiLtJ mice, a model of spontaneous SS development, was increased by the infiltration of Th17, Tfh17, and IL-17-expressing B cells into the salivary glands, resulting in salivary gland cell apoptosis and tissue dysfunction. Since our results demonstrated that RA levels were lower in NOD/ShiLtJ mice, we used RA to treat this SS mouse model to improve SS symptoms. The infiltration of IL-17^+^ cells and the number of α4β7^-^ Th17 cells were decreased in RA-treated mice. We also showed that RA ameliorated imbalance in the Th17/Treg cell population in SS and that IL-17 levels were lower, and Foxp3 levels higher, in RA-treated SS mice. Intestinal damage occurred in NOD/ShiLtJ mice, but was alleviated by RA treatment. RA not only recruits Th17 into the gut but also seems to relieve barrier damage by increasing the Treg cell population.

**Figure 7:**
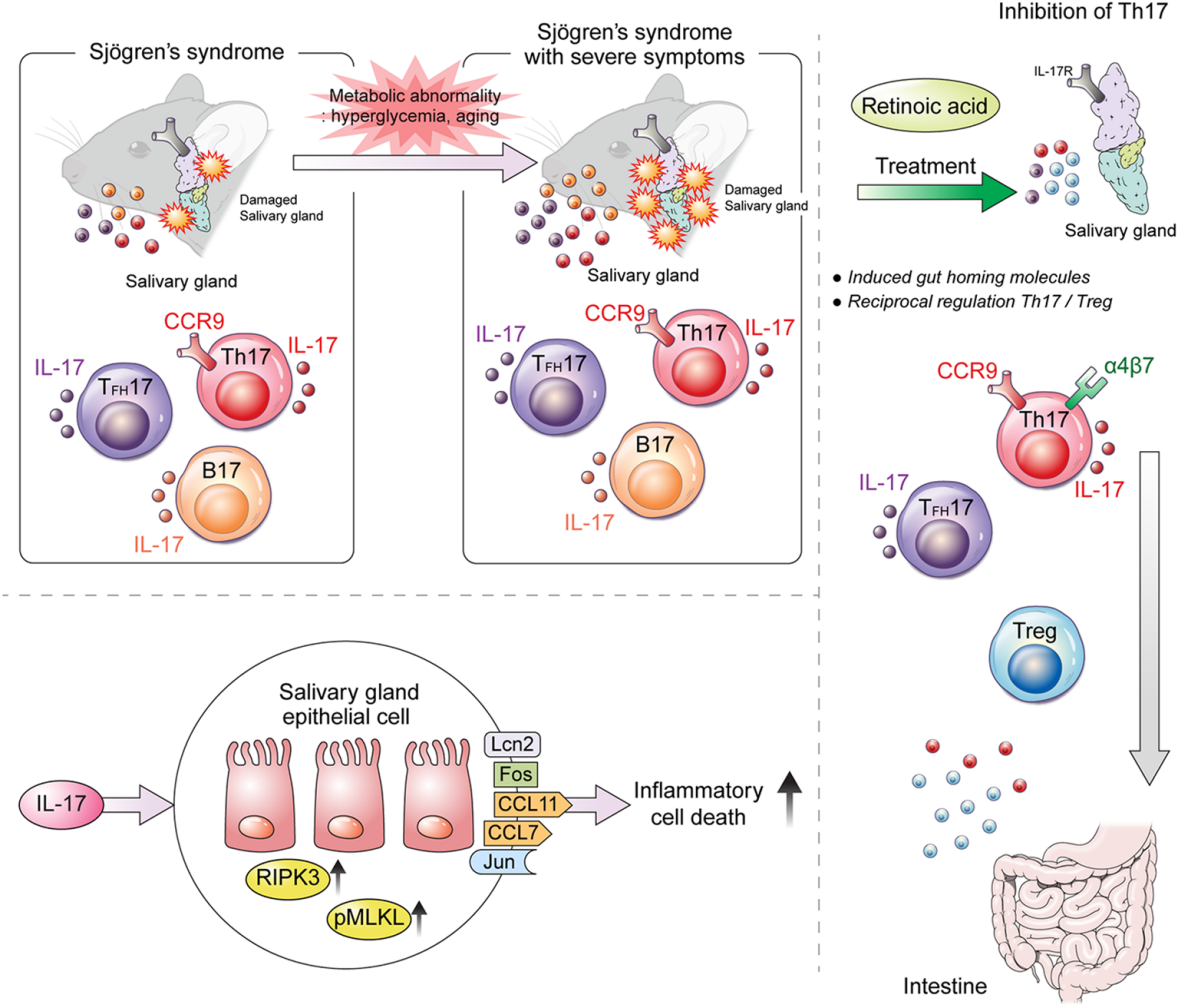
Graphical summary of the findings of the present study. Our study demonstrates that IL-17 and CCR9^+^α4β7^–^ Th17 cells promote salivary gland inflammation and dysfunction in SS. RA ameliorates aging- and metabolic-abnormality-induced salivary gland inflammation by inducing gut-homing CCR9^+^α4β7^+^ Th17 and Treg cells in SS.

The results of the present study suggest that RA treatment may be helpful for relieving and recovering from SS symptoms. Metabolic abnormalities associated with aging exacerbate SS by increasing both the number of IL-17-producing immune cells and their infiltration into salivary glands due to reduced RA levels (**Figure 7**). Our findings suggest that RA may be a key regulator of the development of IL-17-mediated SS.

## Data Availability Statement

The datasets presented in this study can be found in online repositories. The names of the repository/repositories and accession number(s) can be found in the article/**Supplementary Material**.

## Ethics Statement

The studies involving human participants were reviewed and approved by Institutional Review Board of Seoul St. Mary’s Hospital. The patients/participants provided their written informed consent to participate in this study. The animal study was reviewed and approved by Animal Research Ethics Committee of the Catholic University of Korea.

## Author Contributions

S-HH and M-LC: conception and design of study. S-HH, SY, JL, JC, and KL: acquisition data. S-HH, JM, and J-SP: analysis and interpretation of data. S-HH, JM, JW S-KK, S-HP, and M-LC: drafting the article. All authors contributed to the article and approved the submitted version.

## Funding

This work was supported by the National Research Foundation of Korea (NRF) grant funded by the Korea government (MSIT) (No. 2020R1A2C2099615). This research was supported by a grant of the Korea Health Technology R&D Project through the Korea Health Industry Development Institute (KHIDI), funded by the Ministry of Health & Welfare, Republic of Korea (grant number HI20C1496).

## Conflict of Interest

The authors declare that the research was conducted in the absence of any commercial or financial relationships that could be construed as a potential conflict of interest.

## Publisher’s Note

All claims expressed in this article are solely those of the authors and do not necessarily represent those of their affiliated organizations, or those of the publisher, the editors and the reviewers. Any product that may be evaluated in this article, or claim that may be made by its manufacturer, is not guaranteed or endorsed by the publisher.
