# Supplementary material for: IL-17 and CCR9+α4β7– Th17 Cells Promote Salivary Gland Inflammation, Dysfunction, and Cell Death in Sjögren’s Syndrome
Source: Front Immunol. 2021 Sep 1;12:721453. doi: 10.3389/fimmu.2021.721453 (PMC8440850; doi:10.3389/fimmu.2021.721453)
Supplement: Supplementary file 1 [file DataSheet_1.docx]

**IL-17 and CCR9^+^α4β7^–^ Th17 cells promote salivary gland inflammation, dysfunction, and cell death in Sjögren’s syndrome**

Running title: IL-17 and CCR9^+^α4β7^–^ Th17 cells promote Sjögren’s syndrome

Sun-Hee Hwang^1^, Jin Seok Woo^1^, Jeonghyeon Moon^1^, SeungCheon Yang^1^, Jin-Sil Park^1^, JaeSeon Lee^1^, JeongWon Choi^1^, Kun Hee Lee^1,2^, Seung-Ki Kwok^1,3^, Sung-Hwan Park^1,3^, Mi-La Cho^1,4^

^1^The Rheumatism Research Center, Catholic Research Institute of Medical Science, College of Medicine, The Catholic University of Korea, Seoul 06591, Republic of Korea

^2^Department of Biomedicine & Health Sciences, College of Medicine, The Catholic University of Korea, 222, Banpo-daero, Seocho-gu, Seoul, 06591, Republic of Korea

^3^Divison of Rheumatology, Department of Internal Medicine, Seoul St. Mary’s Hospital, College of Medicine, The Catholic University of Korea, Seoul 06591, Republic of Korea

^4^Department of Medical Lifescience, College of Medicine, The Catholic University of Korea, Seoul 06591, Republic of Korea

**Correspondence should be addressed to**

**Sung-Hwan Park**, **M.D., PhD**, Division of Rheumatology, Department of Internal Medicine, School of Medicine, The Catholic University of Korea, Seoul St. Mary’s Hospital, 222 Banpo-Daero, Seocho-gu, Seoul 06591, Republic of Korea (Tel: 82-2-2258-7473, Fax: 82-2-599-3589, E-mail address: [rapark@catholic.ac.kr](mailto:rapark@catholic.ac.kr))

**Mi-La Cho, Ph.D.,** Rheumatism Research Center, Catholic Research Institute of Medical Science, College of Medicine, The Catholic University of Korea, Seoul 137-040, Republic of Korea (Tel: 82-2-2258-7473, Fax: 82-2-2258-7473, E-mail: [iammila@catholic.ac.kr](mailto:iammila@catholic.ac.kr))

**Supplementary Figures and Legends**


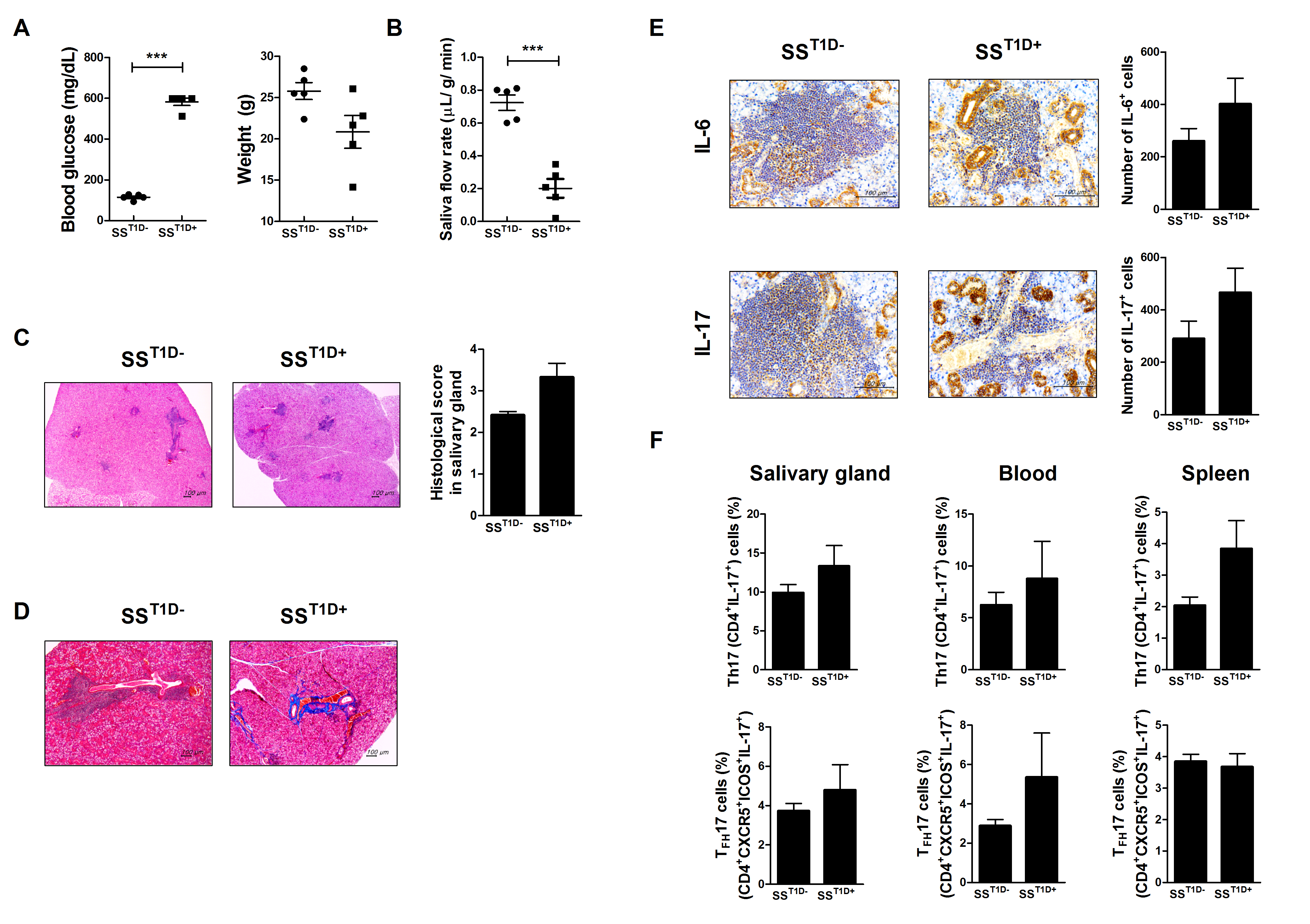


**Supplementary Figure 1. Investigation of Sjögren’s syndrome (SS) and T1D symptoms in NOD/ShiLtJ mice.** **(A)** Bar graphs show blood glucose levels (left) and weights (right) of NOD/ShiLtJ mice with (N=5) or without T1D (N=5). **(B)** Bar graph shows salivary flow rates of NOD/ShiLtJ mice with or without T1D. **(C)** Representative hematoxylin and eosin (H&E)-stained images of salivary glands from NOD/ShiLtJ mice with (N=5) or without T1D (N=5). Bar graph shows average histological scores. Scale bar = 100 µm. **(D)** Representative Masson’s trichrome-stained images of salivary glands from NOD/ShiLtJ mice with (N=5) or without T1D (N=5). Scale bar = 100 µm. **(E)** Representative IL-6- (top) and IL-17- (bottom) stained images of salivary glands from NOD/ShiLtJ mice with (right) (N=5) or without (left) T1D (N=5). Bar graphs show average numbers of IL-6- (top) and IL-17- (bottom) positive cells in salivary gland tissues. **(F)** Bar graphs show Th17 (CD4^+^IL-17^+^, top) and T_FH_17 (CD4^+^CXCR5^+^ICOS^+^IL-17^+^, bottom) cells among cells isolated from salivary glands (left), peripheral blood (center), and spleens (right) of NOD/ShiLtJ mice with (N=5) or without T1D (N=5). Cells were stimulated with phorbol 12-myristate 13-acetate (PMA) and ionomycin for 4 h and GolgiStop for the final 2 h, and then stained with indicated antibodies for flow cytometry analysis. Values are means ± SEM from three independent experiments. *** *p* < 0.001.


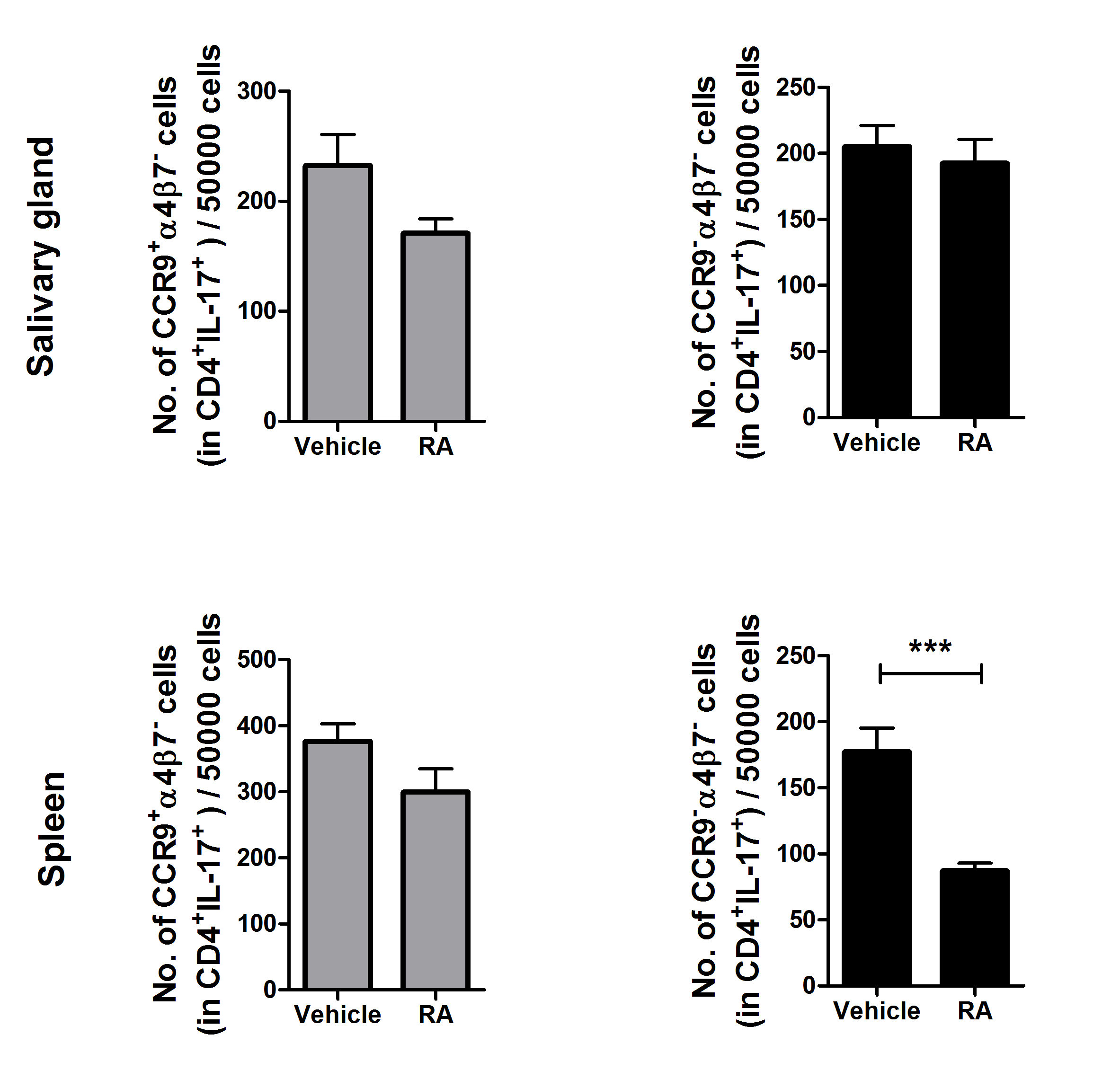


**Supplementary Figure 2. Expression of gut-homing molecules in IL-17-producing cells by following retinoic acid (RA) treatment.** Bar graphs show average numbers of CD4^+^CD17^+^CCR9^+^α4β7^–^ (left) and CD4^+^CD17^+^CCR9^–^α4β7^–^ (right) per 50,000 cells among cells isolated from salivary glands (top) and spleens (bottom) of vehicle- (N=5) and RA-treated NOD/ShiLtJ mice (N=5). Cells were stimulated with PMA and ionomycin for 4 h and GolgiStop for the final 2 h, and then stained with the indicated antibodies for flow cytometry analysis. Values are means ± SEM from three independent experiments. *** *p* < 0.001.
